# Ewing’s Sarcoma of the Prostate Presenting as Back Pain: A Report of a Rare Case and Review of the Literature

**DOI:** 10.7759/cureus.94955

**Published:** 2025-10-19

**Authors:** Kavi Chakravarthi M.G, Latha KVS, A Ravichandran, Yatish M Ellini, Sandhya Sundaram

**Affiliations:** 1 Medical Oncology, Sri Ramachandra Institute of Higher Education and Research, Chennai, IND; 2 Internal Medicine, Venkateshwara Hospitals, Chennai, IND; 3 Pathology, Sri Ramachandra Institute of Higher Education and Research, Chennai, IND

**Keywords:** back pain, extraskeletal ewing's sarcoma, prostate sarcoma, rare case report, small round blue cell tumor, vac-ie chemotherapy

## Abstract

Ewing’s sarcoma is an aggressive small round blue cell tumor typically arising in bone or soft tissues of children and young adults. Primary involvement of the prostate is exceptionally rare, with only a handful of cases reported. The unusual site and nonspecific clinical presentation often delay diagnosis.

We report the case of a 35-year-old male patient who initially presented with progressive lower back pain of two months' duration, without neurological deficits. The pain was accompanied by urinary hesitancy and abdominal discomfort. Imaging revealed a markedly enlarged prostate (13.2 × 11.1 × 18.1 cm) with heterogeneous enhancement, cystic degeneration, and compression of adjacent pelvic structures. PET-CT demonstrated intense fluorodeoxyglucose (FDG) uptake in the prostate and distant metastases involving the lungs and liver. Histopathology of a core biopsy showed sheets of small round blue cells with hyperchromatic nuclei and scant cytoplasm, along with rosette formation. Immunohistochemistry (IHC) was positive for CD99, FLI-1, and NKX2.2, with a high Ki-67 proliferative index (80%). Fluorescence in situ hybridization (FISH) confirmed an EWSR1 gene rearrangement, establishing the diagnosis of Ewing’s sarcoma of the prostate.

The patient was started on multimodality VAC-IE (vincristine, doxorubicin, and cyclophosphamide alternating with ifosfamide and etoposide) chemotherapy. Given the presence of systemic metastases at diagnosis, the prognosis remains guarded, but early initiation of therapy was pursued to achieve disease control.

This case underscores the diagnostic challenge of Ewing’s sarcoma in an unusual site such as the prostate, particularly when presenting with back pain rather than urological complaints. Persistent or unexplained back pain in young adults should prompt consideration of rare malignancies. Comprehensive imaging, histopathology, IHC, and molecular analysis remain indispensable for diagnosis. Early recognition and a multidisciplinary treatment approach are essential to improving outcomes in this aggressive disease.

## Introduction

Ewing’s sarcoma is a highly aggressive small round blue cell tumor that predominantly affects children and young adults. It most commonly arises in long bones, the pelvis, and the axial skeleton, but can also involve soft tissues in what is termed extraosseous Ewing’s sarcoma [1]. Typically, Ewing's sarcoma is characterized by localized pain, swelling, and a lump near the affected bone, along with fatigue, weight loss, and fever spikes, which are worse at night. At a genomic level, translocations involving EWSR1 are a hallmark of Ewing's sarcoma; this gene is responsible for making the EWS protein. EWS is a multifunctional protein involved in gene expression, RNA processing, and cell signaling.

Primary occurrence of Ewing's sarcoma within the genitourinary tract is exceedingly rare, and among these, prostatic origin is an especially uncommon entity. Since its first description, only a limited number of cases of primary prostatic Ewing’s sarcoma/primitive neuroectodermal tumor (PNET) have been reported in the literature [2]. The diagnostic challenge is compounded by the nonspecific clinical presentation. Prostatic Ewing’s sarcoma may manifest with lower urinary tract symptoms such as frequency, urgency, dysuria, or urinary retention, but atypical complaints, including abdominal pain, constipation, or back pain, can also occur [3]. The rarity of this disease and its overlapping symptomatology with more common urological disorders often result in delayed recognition. Radiologically, these tumors appear as large, heterogeneous masses that can infiltrate and compress adjacent pelvic organs, which was evident in our case [4]. Definitive diagnosis, however, requires histopathological examination, immunohistochemical profiling, and increasingly molecular confirmation through detection of EWSR1 translocation. Immunohistochemistry (IHC) demonstrating positivity for CD99, FLI-1, and NKX2.2 is strongly supportive, while a high proliferative index further underscores the aggressive biology of the tumor [5]. Therapeutically, the mainstay of management involves systemic chemotherapy, typically the VAC-IE regimen (vincristine, doxorubicin, and cyclophosphamide alternating with ifosfamide and etoposide), often combined with surgery or radiotherapy where feasible. However, due to advanced disease at presentation and the aggressive natural history, prognosis remains poor in many cases [6].

This report describes a rare case of a 35-year-old male patient presenting with persistent back pain as the initial symptom of prostatic Ewing’s sarcoma. The case highlights the importance of maintaining a broad differential diagnosis for atypical back pain, the pivotal role of multimodal diagnostic evaluation, and the need for a multidisciplinary approach in managing such rare and aggressive malignancies.

## Case presentation

Patient demographics

A 35-year-old previously healthy male presented with progressive lower back pain persisting for two months. The pain was dull, non-radiating, and associated with reduced mobility, significantly affecting daily activities, grade 2 or 3 as per the Graded Chronic Pain Scale (GCPS). Over time, he developed urinary hesitancy and a sensation of incomplete bladder emptying, along with mild lower abdominal discomfort, sometimes associated with a sharp, cramping-like pain. There was no history of hematuria, weight loss, fever, or prior urological illness.

Clinical findings

On examination, there was localized tenderness over the lumbosacral spine without any motor or sensory neurological deficits. Digital rectal examination revealed a markedly enlarged, firm, and irregular prostate mass, raising suspicion of an underlying pelvic malignancy. Routine laboratory investigations were largely unremarkable, except for mild anemia. Serum prostate-specific antigen (PSA) was within normal limits, which made adenocarcinoma of the prostate less likely.

Diagnostic workup

Imaging

An MRI of the spine demonstrated diffuse hypointense signals on T1/T2 imaging involving multiple vertebral bodies and posterior elements, without evidence of intraspinal extension.

Contrast-enhanced CT of the pelvis revealed a grossly enlarged prostate gland measuring 13.2 × 11.1 × 18.1 cm (volume ~1300 cc). The mass displayed heterogeneous enhancement with multiple cystic areas and was compressing the urinary bladder and rectum, with evidence of distal ureteric infiltration.

**Figure 1:**
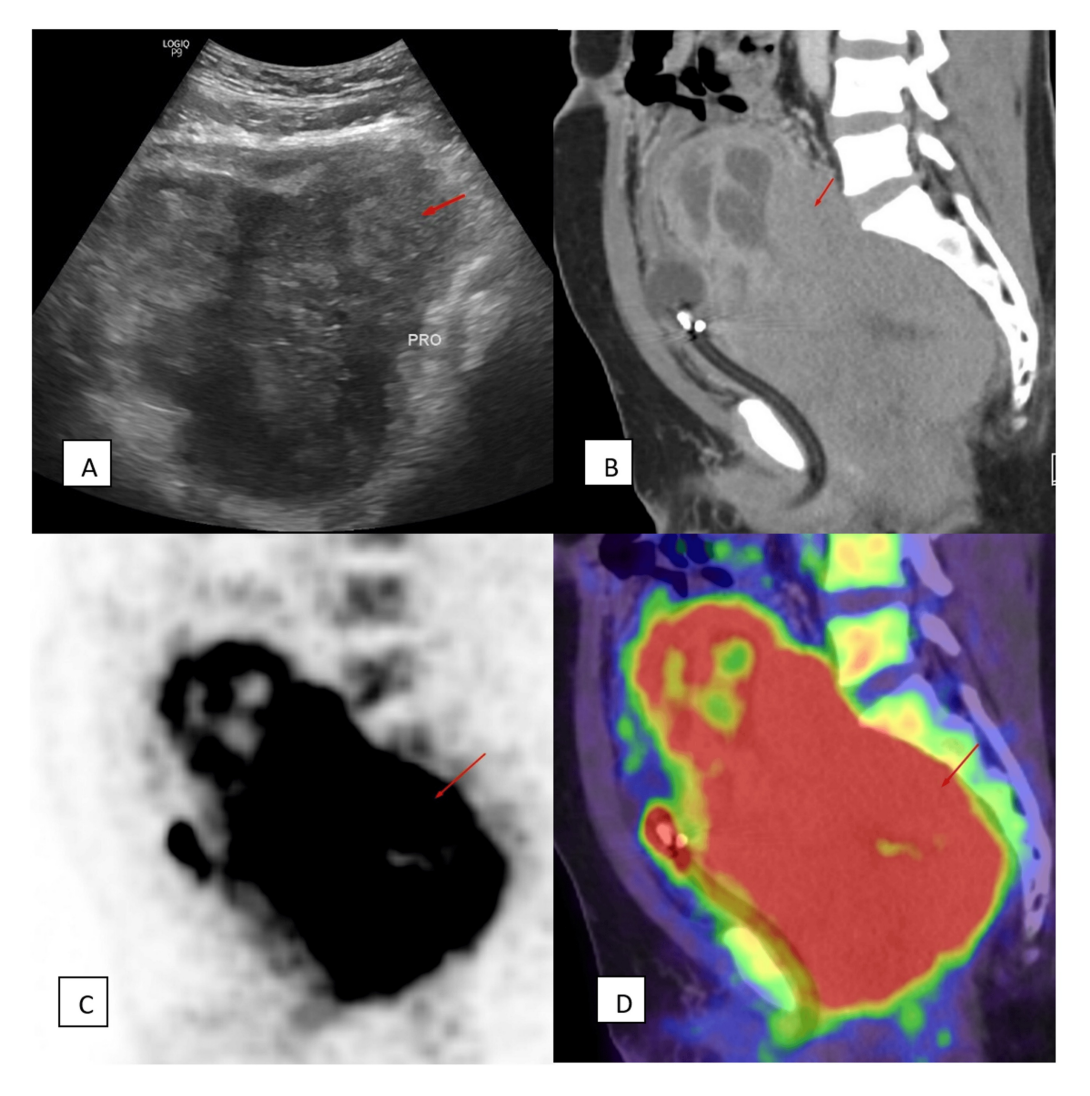
(A) Ultrasound, (B) PET-CT images of the prostate mass demonstrating heterogeneous echotexture, (C) diffuse post-contrast enhancement, and (D) avid FDG uptake. Ultrasound shows a grossly enlarged prostate with heterogeneous echotexture (A). Grossly enlarged prostate with heterogeneous post-contrast enhancement (C). On PET-CT images, the enlarged prostate shows diffuse avid FDG uptake (D). FGD: fluorodeoxyglucose

The PET-CT showed an intensely fluorodeoxyglucose (FDG)-avid pelvic lesion (maximum standardized uptake value (SUV max 16.92)) corresponding to the enlarged prostate (Figures 1A-1D), with multiple pulmonary nodules and liver metastases consistent with systemic spread. 

Histopathology and IHC

**Figure 2:**
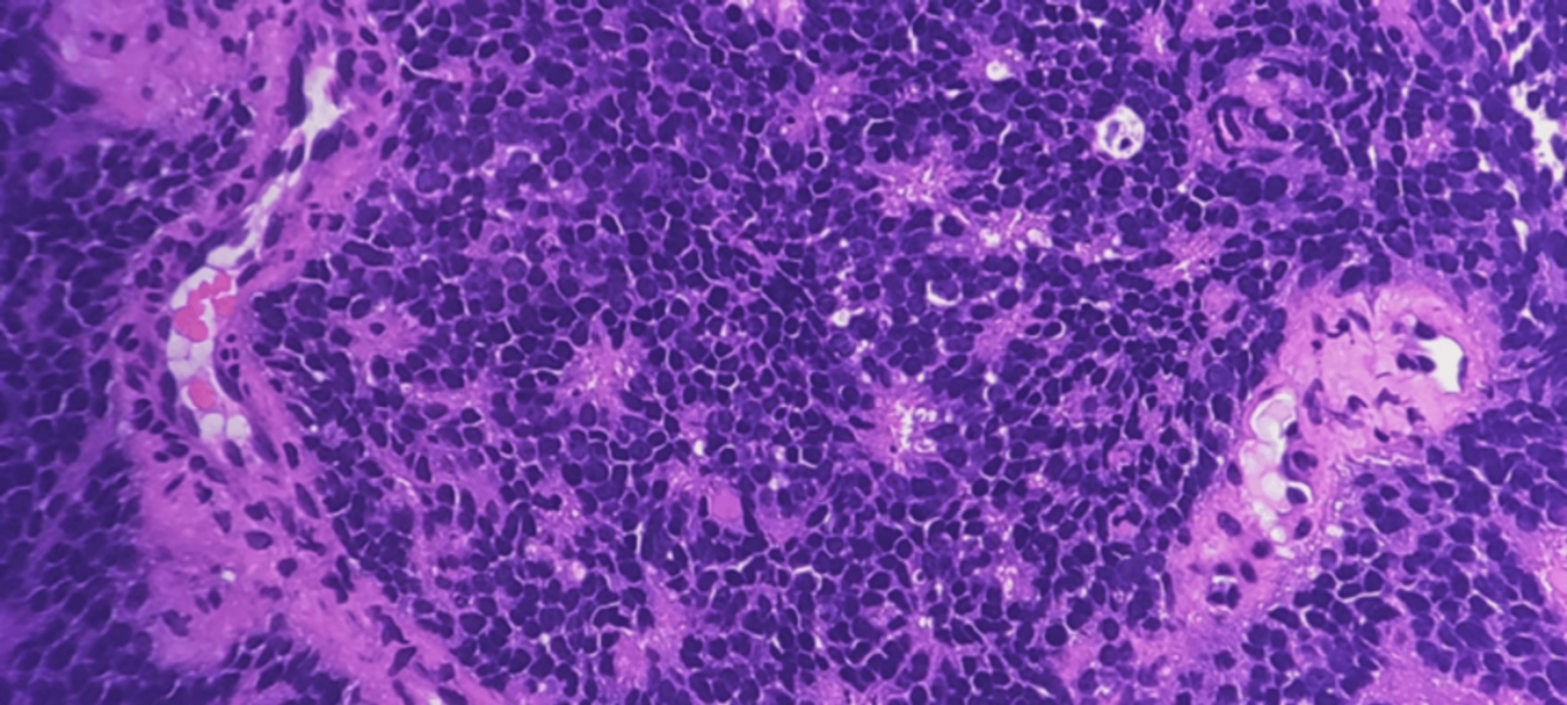
Histopathology and immunohistochemistry (IHC) H&E showing small blue cells with hyperchromatic nuclei and scant cytoplasm. Rosette formation seen; IHC positive for NKX2.2

A transrectal ultrasound-guided core needle biopsy of the prostate was performed. Histopathology revealed sheets of small, round blue cells with hyperchromatic nuclei, scant cytoplasm, and occasional rosette formation (Figure 2).

IHC was diffusely positive for CD99, FLI-1, and NKX2.2, with a Ki-67 proliferation index of approximately 80%. Fluorescence in situ hybridization (FISH) confirmed EWSR1 gene rearrangement, establishing the diagnosis of Ewing’s sarcoma of the prostate.

Management

Given the advanced stage of disease with pulmonary and hepatic metastases at presentation, a curative surgical approach was not feasible. The patient was initiated on multimodality systemic chemotherapy using the VAC-IE regimen. Supportive care measures were also instituted, including pain management and nutritional support. The patient is currently under close follow-up to assess treatment response.

## Discussion

Ewing’s sarcoma belongs to the family of small round blue cell tumors characterized by specific chromosomal translocations, most commonly involving the EWSR1 gene. While the majority of cases arise in bone or soft tissues of the extremities and axial skeleton, extraosseous Ewing’s sarcoma accounts for approximately 15%-20% of cases. Within the genitourinary tract, reported sites include the kidney, bladder, and penis, but primary involvement of the prostate remains exceptionally rare [7,8].

Rarity of prostatic Ewing’s sarcoma

Fewer than 50 cases of primary prostatic Ewing’s sarcoma or PNET have been reported worldwide. The majority of these cases occur in young to middle-aged men, typically between 20 and 40 years of age. Clinical presentation is often nonspecific, mimicking benign prostatic hyperplasia, prostatitis, or adenocarcinoma of the prostate. Lower urinary tract symptoms are the most common, while hematuria, pelvic pain, and acute urinary retention have also been described. Back pain as the predominant symptom, as in our case, is exceedingly uncommon and may contribute to diagnostic delays [9,10].

Diagnostic considerations

Radiological findings typically reveal a large, heterogeneous pelvic mass with cystic or necrotic components and mass effect on surrounding organs. However, imaging alone cannot reliably distinguish Ewing’s sarcoma from other malignant prostate tumors. Histopathology remains essential, demonstrating the characteristic small round blue cells with scant cytoplasm and occasional rosette formation. IHC provides further specificity, with diffuse membranous positivity for CD99 and nuclear positivity for FLI-1 and NKX2.2 considered diagnostic hallmarks. The presence of an EWSR1 rearrangement confirmed by molecular studies such as FISH or reverse transcription polymerase chain reaction (RT-PCR) clinches the diagnosis [11,12].

In our case, all diagnostic modalities contributed to establishing a definitive diagnosis: radiological evidence of a massive FDG-avid prostate lesion with metastases, histology showing small round blue cells, IHC positivity for CD99, FLI-1, and NKX2.2, and molecular confirmation of EWSR1 rearrangement.

Treatment and prognosis

Treatment of Ewing’s sarcoma generally involves multimodal therapy comprising systemic chemotherapy, local control (surgery and/or radiotherapy), and supportive care. The VAC-IE regimen is the current standard of care and has shown significant survival benefit in both localized and metastatic disease. Surgical resection or radical prostatectomy may be considered for localized tumors, often followed by chemotherapy and/or radiotherapy. However, most cases present at an advanced stage, limiting curative surgical options [13].

The prognosis of prostatic Ewing’s sarcoma remains poor, particularly in patients with metastatic disease at diagnosis. Reported five-year survival rates in localized Ewing’s sarcoma can approach 60%-70%, but in metastatic cases, survival drops significantly, often below 30%. Our patient presented with pulmonary and hepatic metastases, reflecting the aggressive nature of the disease and underscoring the importance of early recognition [14].

Literature context and case significance

Wu et al. described a similar case of Ewing’s sarcoma of the prostate, highlighting diagnostic pitfalls and the role of chemotherapy [15]. Kumar et al. also reported a prostatic PNET with overlapping histological features [16].

In comparison, our case is notable for its unusual primary presentation as back pain rather than urinary symptoms. This emphasizes the importance of including rare malignancies in the differential diagnosis of unexplained back pain, particularly in younger patients (Table 1).

**Table 1:** Comparison of our case with similar published literature

Author/Year	Age (years)	Presenting Symptoms	Tumor Size/Imaging Findings	Diagnostic Confirmation	Treatment Given	Outcome/Follow-up	Notable Features
Current case (2025)	35	Progressive lower back pain, urinary hesitancy, mild abdominal discomfort	Prostate 13.2 × 11.1 × 18.1 cm; heterogeneous enhancement; PET-CT: lung & liver metastases	Histology: small round blue cells; immunohistochemistry (IHC): CD99, FLI-1, NKX2.2 positive; fluorescence in situ hybridization (FISH): EWSR1 rearrangement	VAC-IE chemotherapy (vincristine, doxorubicin, cyclophosphamide alternating with ifosfamide, etoposide)	Under treatment; guarded prognosis due to systemic metastases	Unusual primary presentation as back pain
Funahashi et al., 2009 [14]	33	Lower urinary tract symptoms (LUTS)	Large heterogeneous prostatic mass on imaging	Histology + IHC for CD99	Radical prostatectomy + adjuvant chemotherapy	Alive with no evidence of disease at 12 months	Early localized disease permitted surgery
Wu et al., 2013 [15]	20s (exact age not stated in excerpt)	Urinary obstruction	Large pelvic mass	Histology + IHC + molecular confirmation	VAC-IE chemotherapy	Partial response; long-term outcome not specified	Highlighted the importance of early systemic therapy
Esch et al., 2016 [3]	33	LUTS	Large prostatic mass with cystic changes	Histology + IHC (CD99)	Chemotherapy + radiotherapy	Alive and disease-free at 18 months	Typical LUTS presentation; good early outcome

Key learning points

Atypical Presentation

Persistent back pain in young adults should not be dismissed and warrants a comprehensive evaluation, particularly when associated with subtle urinary symptoms.

Multimodal Diagnosis

Accurate diagnosis of Ewing’s sarcoma requires a combination of imaging, histopathology, IHC, and molecular studies.

Aggressive Biology

Prostatic Ewing’s sarcoma often presents at an advanced stage, necessitating systemic chemotherapy as the cornerstone of management.

Multidisciplinary Care

Optimal management requires collaboration between urologists, oncologists, pathologists, and radiologists to tailor individualized treatment.

## Conclusions

Primary Ewing’s sarcoma of the prostate is an exceedingly rare malignancy, often presenting with nonspecific or atypical symptoms that delay diagnosis. Our case illustrates an unusual presentation where persistent back pain, rather than predominant urinary complaints, was the initial clinical feature. This highlights the importance of maintaining a broad differential diagnosis when evaluating young patients with unexplained musculoskeletal or pelvic symptoms. Accurate diagnosis relies on a multimodal approach: combining advanced imaging, histopathological evaluation, IHC, and molecular studies. Early recognition is critical given the aggressive nature of this tumor and its tendency to present with systemic metastases. Although the VAC-IE chemotherapy regimen remains the standard of care, outcomes in advanced cases are guarded, underscoring the need for continued research into novel therapeutic strategies.

Ultimately, this case contributes to the limited literature on prostatic Ewing’s sarcoma, reinforces the role of multidisciplinary evaluation, and emphasizes vigilance for rare malignancies in atypical clinical scenarios.
